# 3D multidetector CT angiographic evaluation of intralobular bronchopulmonary sequestration

**DOI:** 10.4103/0971-9261.69144

**Published:** 2010

**Authors:** Ruchira Marwah, Jaykumar R. Nair, Arbinder Singal, Inder Talwar

**Affiliations:** Department of Radiodiagnosis and Imaging, Bombay Hospital and Research Centre, Mumbai, India; 1 Department of Pediatric Surgery, MGM Hospital, Vashi, New Bombay, India

**Keywords:** 3D CT angiography, anomalous venous drainage, intralobar sequestration, maximum intensity projection, volume rendering technique

## Abstract

We report a case of intralobar pulmonary sequestration with special emphasis on computed tomography (CT) angiography in determining the arterial supply and venous drainage, thus providing a detailed knowledge of the vasculature, which is of vital importance in surgery. The 3D volume rendering technique and maximum intensity projection images provide the vascular road map for the surgeon.

## INTRODUCTION

Pulmonary sequestration is a congenital malformation in which an area of dysplastic and nonfunctioning lung parenchyma is present without a normal connection to the tracheobronchial tree and the pulmonary arteries.[1] It receives its vascular supply from a systemic artery. It is anatomically classified as intralobar and extralobar types. Intralobar pulmonary sequestration is located within the visceral pleura and is surrounded by normal lung, whereas extralobar pulmonary sequestration is separated from the lung by a pleural envelope. The venous drainage of intralobar pulmonary sequestration is generally via the pulmonary veins, whereas extralobar usually has a systemic venous return.[2]

Here we report a case of intralobar sequestration with special emphasis on computed tomography (CT) angiography for preoperative evaluation. To the best of our knowledge, CT angiography using a 64-slice CT scanner has not been reported for preoperative evaluation of pulmonary sequestration.

## CASE REPORT

**Figure 1:**
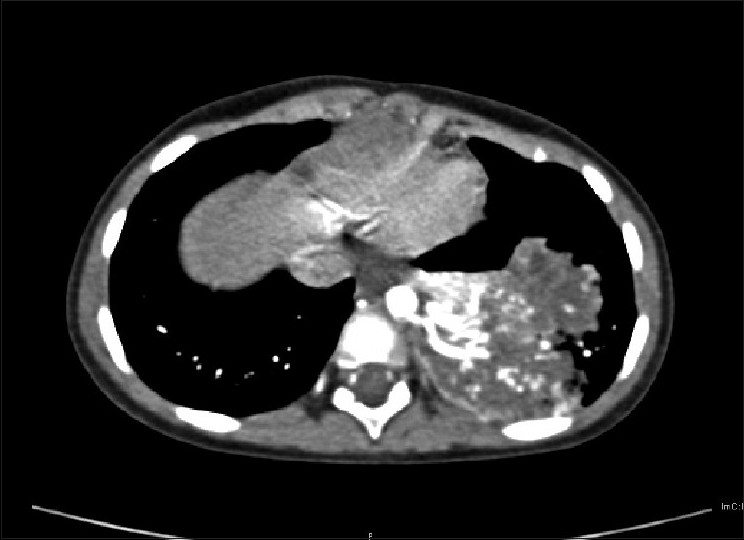
Axial CT angiographic image showing anomalous vessels arising from the descending aorta and supplying the enhancing left lower lobe sequestered lung segment

**Figure 2:**
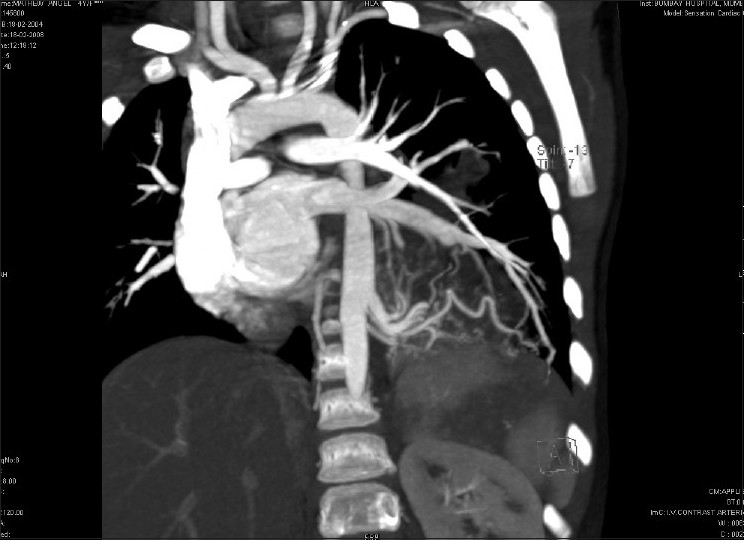
Coronal reformatted image of the venous phase showing multiple veins from the left lower lobe sequestered lung segment draining into the left inferior pulmonary vein and eventually into the left atrium

**Figure 3:**
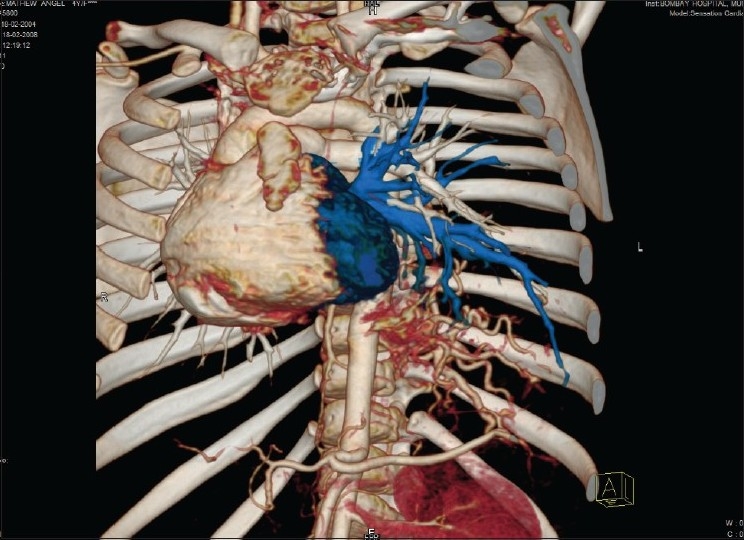
3D volume rendered image showing anomalous arteries arising from the aorta supplying the left lower lobe of lung and anomalous veins draining into the left inferior pulmonary vein

A 4-year-old girl presented with repeated episodes of chest infection. Sequential chest radiographs showed an area of nonresolving consolidation in the left lower lobe. She was referred to the radiology department for CT angiography based on the clinical suspicion of pulmonary sequestration. The patient underwent multidetector CT on 64-slice state-of-the-art Siemens scanner. The examination was performed at 150 mA and 120 kV. Plain scan of the chest was performed from the level of thoracic inlet to just below the inferior pole of the kidney. Non-ionic iodinated contrast 50 mL was given at the dose of 2 mL/kg of body weight for the angiographic study. Using the bolus tracking method, the angiogram was done by an automatic trigger system with a density of 100 HU in the ascending aorta. CT scan was acquired during quiet respiration without sedation. Images were processed with standard soft tissue settings (300 window width [WW], 40 window length [WL]) and lung settings (1200 WW, -600 WL). Axial CT images showed a solid heterogenously enhancing mass located in the left lower lobe, above the diaphragm. On angiography, three anomalous vessels arising from the descending aorta were seen supplying the enhancing mass lesion on the arterial phase [Figure 1]. Multiple veins were seen draining the mass into the left inferior pulmonary vein and eventually into the left atrium on the venous phase [Figure 2]. Evaluation of the origin as well as the entire course was difficult with axial images alone. Hence further evaluation was done by processing the images and obtaining 3D volume rendered and maximum intensity projection images in different anatomic planes using the Wizard workstation [Figure 3]. Based on the radiologic findings, the pediatric surgeon did a thoracotomy and resected the sequestered lower lobe lung segment. Our findings were confirmed intraoperatively. Postoperative chest radiograph revealed resolution of left lower zone consolidation. Along with sequestration, another anomaly (ectopic right kidney in the pelvis) was picked up during the course of the study.

## DISCUSSION

Pulmonary sequestration is a rare congenital anomaly among all congenital malformations (0.5%–6.4%).[3] Previously, a catheter angiography was performed to diagnose sequestration and demonstrate its blood supply. However, now spiral CT angiography offers a less invasive means of demonstrating the anomalies.[4] Visualization of the anomalous arteries and veins is of great significance in making the diagnosis of pulmonary sequestration and differentiating it from other lung parenchymal abnormalities. The ability of CT angiography to simultaneously image the arterial supply, venous drainage, and parenchymal changes in a single examination makes it the imaging modality of choice.[5] With the information available, intralobar sequestration can be distinguished from extralobar sequestration. Most extralobar sequestrations require lobectomy or segmentectomy of the involved lung, whereas the sequestered segment can be removed without resection of the normal lung tissue in extralobar sequestration. Furthermore, optimally defining an infradiaphragmatic anomalous vessel is important because it necessitates transabdominal surgery rather than transthoracic intervention.[6] 3D VRT, multiplanar reconstruction, and MIP images further aid the surgeon in detailed evaluation, giving a vascular road map. Preoperative identification of anomalous venous drainage in a sequestered segment can prevent massive intraoperative hemorrhage due to accidental transection of an unanticipated vessel.[7] Extralobar sequestration is rare and is typically associated with other inborn abnormalities. Intralobar sequestration is infrequently associated with other developmental anomalies.[8] The abnormal sequestered segment was successfully excised by the surgeon based on the vascular road map provided by the CT angiography images.
